# The Evolving Landscape of NMR Structural Elucidation

**DOI:** 10.3390/molecules31050888

**Published:** 2026-03-07

**Authors:** Josep Saurí

**Affiliations:** Organic & Pharmaceutical Chemistry Department, Institut Químic de Sarrià, Universitat Ramon Llull, Via Augusta 390, 08017 Barcelona, Catalonia, Spain; josep.sauri@iqs.url.edu

**Keywords:** NMR spectroscopy, structure elucidation

## Abstract

Nuclear Magnetic Resonance (NMR) spectroscopy has long been a cornerstone in the structural elucidation of molecules, offering unique insights into atomic-level connectivity, conformation, and dynamics. Over the past decades, methodological and technological advances have significantly expanded its capabilities and applications. This manuscript charts the evolution of NMR from classical 1D/2D experiments to modern methods empowered by ultrahigh magnetic fields, cryogenic probes, non-uniform sampling, new methodologies, and hyperpolarization. We emphasize the growing synergy between experiment and computation, where automated analysis, quantum chemical calculations, and machine learning are dramatically enhancing the accuracy and efficiency of structure determination. We also highlight NMR’s broadening scope in areas ranging from complex mixtures and natural products to biomolecular and materials science.

## 1. Introduction

Nuclear Magnetic Resonance (NMR) spectroscopy occupies a unique place among the analytical techniques available for the characterization and structural elucidation of matter. Since its discovery in the mid-twentieth century, NMR has evolved into one of the most versatile methods for chemists, biologists, and materials scientists. Its unique strength lies in its ability to provide atomic-level insights into molecular structure, conformation, and dynamics in a non-destructive and highly versatile manner. Unlike diffraction techniques, which require crystalline or otherwise ordered samples, or mass spectrometry, which relies on ionizing molecules into the gas phase and yields structural information through fragmentation or ion-mobility behavior, NMR offers a window into molecules as they exist in solution, solid state, or even within living systems.

Over the past several decades, NMR has repeatedly demonstrated its central role in structural elucidation, enabling chemists to move beyond elemental analysis and degradation strategies toward direct spectroscopic determination of molecular architecture [1]. The advent of multidimensional NMR opened the door to high-resolution studies of proteins, nucleic acids, and their complexes in solution, complementing and at times surpassing crystallography in capturing conformational ensembles and dynamics. In materials science, solid-state NMR has matured into a powerful approach to investigate amorphous, crystalline, and hybrid systems that defy characterization by diffraction alone [2,3].

The sustained impact of NMR stems from two closely connected streams of progress. On the one hand, advances in instrumentation—stronger magnets, cryogenic probe technology, hyperpolarization, and improved electronics—have steadily pushed the boundaries of sensitivity and resolution. On the other hand, methodological innovations such as multidimensional correlation experiments, sophisticated pulse sequence developments, and fast and ultra-fast sampling approaches, amongst several others, have multiplied the kinds of structural information accessible to the spectroscopist. Together, these technological and methodological pathways have expanded NMR’s scope far beyond the conventional one-dimensional experiments familiar to generations of chemists.

A third, equally transformative development has been the integration of NMR data with computation. Early in the history of the field, NMR interpretation relied almost exclusively on manual analysis, where spectra were deciphered peak by peak by experts with deep experience. Today, computational tools ranging from density functional theory (DFT) calculations of chemical shifts and spin–spin couplings to probabilistic structure elucidation algorithms and machine learning-based predictors provide unprecedented accuracy and automation and have collectively reshaped the practice of NMR analysis [4]. This convergence of experiment and computation has created a synergistic framework in which NMR data are no longer interpreted in isolation but rather in dialog with large-scale calculations, structural databases, and statistical learning.

As we look at the present and future, it is evident that NMR is undergoing a profound transformation. Its role in small-molecule structure elucidation remains indispensable, but the scope is expanding toward ever more complex systems: natural product mixtures, metabolomes, intrinsically disordered proteins, supramolecular assemblies, and functional materials. At the same time, the traditional boundaries between NMR, mass spectrometry, and structural biology techniques such as cryogenic electron microscopy (cryo-EM) and X-ray crystallography are blurring. Increasingly, NMR contributes as part of integrative approaches that combine multiple modalities, each compensating for the limitations of the others.

This manuscript aims to chart this evolving landscape of NMR structural elucidation. We begin by revisiting the historical foundations and classical methods that established NMR as a cornerstone of molecular science. We then trace technological and methodological advances that have dramatically expanded its reach, from ultrahigh-field magnets and NMR pulse sequence development to non-uniform sampling and hyperpolarization. Special attention is given to the growing synergy between NMR and computation, highlighting how automated analysis, quantum chemical predictions, and machine learning are transforming the practice of structure determination. Finally, we explore emerging applications across diverse domains, from chemistry and biology to materials science, and reflect on the challenges and opportunities that will shape the future of NMR.

## 2. The Classical Foundations of NMR Structural Elucidation

The foundations of NMR structure elucidation rest on a series of conceptual and technical milestones achieved during the 1950s to 1980s. The introduction of Fourier transform NMR by Ernst in 1966 transformed signal acquisition and processing, replacing continuous-wave detection with a pulsed excitation scheme that enabled simultaneous observation of all resonances [5]. This innovation was the gateway to multi-dimensional NMR spectroscopy and earned Ernst the Nobel Prize in Chemistry in 1991.

During this period, chemists refined the interpretation of spin–spin couplings (*J*-couplings) and chemical shifts, which remain the most direct indicators of connectivity and local environment. The development of decoupling, selective excitation, and relaxation editing further enhanced the analytical power of 1D spectra.

The next breakthrough came with two-dimensional (2D) NMR. The COSY (Correlation Spectroscopy) experiment established spin–spin connectivities through scalar couplings, while the NOESY (Nuclear Overhauser Effect Spectroscopy) and ROESY (Rotating-frame Overhauser Effect Spectroscopy) experiments provided information on spatial proximities. Heteronuclear correlation methods such as HSQC (Heteronuclear Single Quantum Coherence) and HMBC (Heteronuclear Multiple Bond Correlation) expanded these concepts across multiple nuclei, forming the backbone of modern molecular structure elucidation. By the late 1980s, the combination of 2D NMR and isotopic labeling enabled complete structural characterization of proteins in solution [6], a feat that earned Wüthrich the Nobel Prize in Chemistry in 2002. These developments defined what might be called the “classical era” of NMR, establishing the conceptual and methodological basis upon which all subsequent advances have been built.

### 2.1. Basic Principles

At the heart of Nuclear Magnetic Resonance lies the nuclear spin, a quantum property of many nuclei that endows them with a magnetic moment. When placed in an external magnetic field, spins adopt discrete energy levels whose populations are governed by the Boltzmann distribution. Radiofrequency (RF) pulses can induce transitions between these levels, and the resulting oscillating magnetic fields are detected as free induction decays (FIDs). Fourier transformation converts these time-domain signals into frequency-domain spectra, in which resonances are dispersed according to the nuclear environment. However, because the Boltzmann population differences between the nuclear spin states are exceedingly small—even at high magnetic fields—the net macroscopic magnetization available for detection is tiny. This intrinsically weak polarization is the fundamental reason for the low sensitivity of NMR, motivating the development of numerous signal-enhancement strategies that will be covered in the next sections.

Three central observables form the basis of structural elucidation by NMR: chemical shifts, scalar (*J*) couplings, and relaxation properties. The chemical shift reflects the shielding or deshielding of a nucleus by the surrounding electron distribution and is highly sensitive to functional groups, hybridization, and local geometry. Scalar couplings arise from through-bond electron-mediated interactions and provide information on connectivity and stereochemistry. Relaxation parameters constitute the key physical foundation of the Nuclear Overhauser Effect (NOE), since the phenomenon arises from cross-relaxation mediated by dipole–dipole interactions. Although they are often discussed primarily in the context of molecular dynamics, relaxation parameters also encode information about molecular size and overall tumbling, providing a valuable means of distinguishing between monomeric and aggregated states.

### 2.2. Early 1D Methods

The first decades of NMR structural analysis were dominated by one-dimensional (1D) proton spectra. These spectra provided direct access to hydrogen count, chemical environments, and coupling patterns. Proton decoupling and off-resonance decoupling methods later extended the reach of NMR to heteronuclei, most notably ^13^C, whose low natural abundance and sensitivity initially limited their use. The introduction of proton-decoupled ^13^C spectra in the 1960s was a turning point, allowing chemists to determine carbon skeletons with unprecedented clarity. Refinements such as DEPT (Distortionless Enhancement by Polarization Transfer) [7] experiments brought further structural power by distinguishing between CH, CH_2_, and CH_3_ groups, while selective decoupling experiments allowed chemists to probe specific couplings in detail. Though primitive by modern standards, these 1D approaches laid the conceptual foundations of NMR as a tool for deducing connectivity and functional group placement.

### 2.3. The Rise of 2D NMR

The 1970s marked a transformative turning point in NMR spectroscopy with the emergence of two-dimensional (2D) NMR, first proposed by Jean Jeener in 1971 and later implemented and generalized by Richard R. Ernst and colleagues [8]. This conceptual leap—from recording a spectrum along a single frequency axis to correlating magnetization along two independent evolution periods—redefined what information could be extracted from NMR data. By distributing spectral features across a second frequency dimension, 2D NMR overcame the severe spectral congestion inherent to one-dimensional experiments, enabling chemists to resolve overlapping resonances and directly observe scalar and dipolar interactions that were previously obscured.

At its core, 2D NMR encodes spin interactions through systematic variation in an indirect evolution time (t_1_) followed by Fourier transformation along both the t_1_ and t_2_ axes. The resulting two-dimensional maps provide a rich and intuitive representation of connectivities, couplings, and spatial relationships within a molecule, dramatically enhancing the structural and dynamical insight available from NMR.

**Figure 1:**
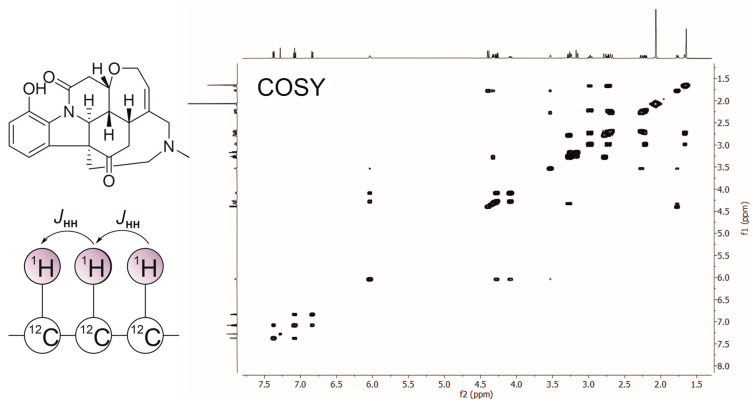
Two-dimensional ^1^H–^1^H COSY NMR spectrum of the alkaloid vomicine. Diagonal peaks correspond to individual proton resonances, while off-diagonal cross-peaks indicate scalar (*J*_HH_) couplings between neighboring protons, as illustrated in the coupling schematic. The spectrum offers key spin–spin correlations used to establish proton connectivity within the molecule.

A suite of now-classical experiments rapidly emerged in subsequent years, each addressing a different aspect of molecular structure. COSY [8] was the first widely adopted 2D experiment, revealing homonuclear through-bond scalar couplings between spins that are 2–3 bonds apart (Figure 1). Its cross-peaks enabled chemists to trace connectivity through proton spin systems, forming the backbone of sequence-specific assignment in small molecules and peptides.

NOESY [9,10] provided access to through-space dipolar interactions, allowing for the measurement of internuclear distances. These distance restraints became the foundational input for three-dimensional structure determination of biomolecules and remain central to modern structural NMR.

**Figure 2:**
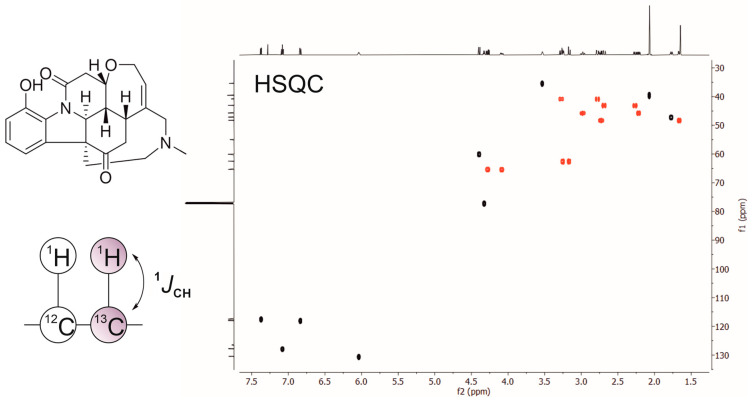
Two-dimensional ^1^H–^13^C HSQC NMR with multiplicity editing spectrum of the alkaloid vomicine. Each cross-peak represents a one-bond heteronuclear coupling (^1^*J*_CH_) between directly attached proton–carbon pairs, as illustrated in the schematic. CH/CH_3_ and CH_2_ carbons have opposite phases, with black signals corresponding to CH and CH_3_ carbons and red signals to CH_2_ carbons.

The HSQC [11] and HMQC (Heteronuclear Multiple Quantum Coherence) [12] methods correlate protons with directly bonded heteronuclei such as ^13^C or ^15^N, drastically simplifying resonance assignment by “spreading out” the spectrum into heteronuclear dimensions. ^1^H-^13^C HSQC, in particular, became a workhorse experiment for organic structure elucidation with features such as multiplicity editing—which allows for differentiation between different types of carbon groups based on the number of attached protons—easily incorporated (Figure 2). Meanwhile, ^1^H-^15^N HSQC became indispensable for biomolecular NMR.

**Figure 3:**
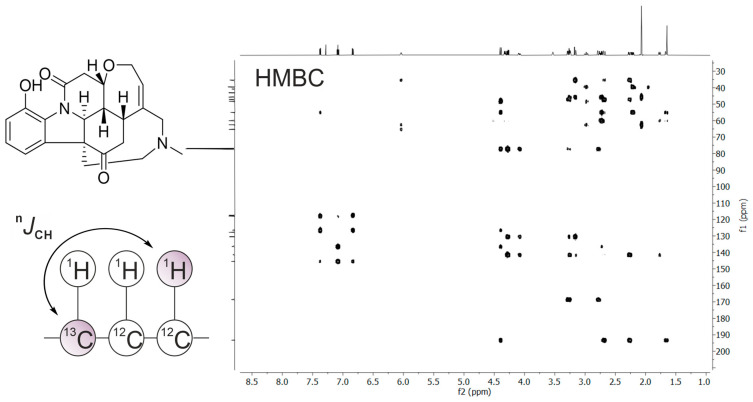
Two-dimensional ^1^H–^13^C HMBC NMR spectrum of the alkaloid vomicine. Long-range heteronuclear correlations (^n^*J*_CH_, typically 2–3 bonds) are observed as cross-peaks between protons and remote carbon atoms, as shown in the schematic. These correlations provide key connectivity information for assembling the carbon skeleton and confirming the positions of proton–carbon linkages across the molecule.

The HMBC experiment [13] allowed for detecting heteronuclear couplings across two or three bonds, enabling chemists to “bridge” segments of molecular frameworks and making it a critical experiment for assembling larger carbon skeletons—especially in synthetic organic chemistry and natural product structure determination. HMBC has solidified itself as one of the most powerful and routinely applied long-range correlation experiments in modern NMR spectroscopy, complementing other 2D methods and forming an essential component of the spectroscopist’s toolkit for unveiling molecular structure (Figure 3).

Beyond simply adding an extra axis to reduce overlap, the triad HSQC-COSY-HMBC—complemented with NOESY—introduced distinct types of connectivity that resolved crowding both geometrically (through an extra dimension) and conceptually (through orthogonal structural information). Collectively, these techniques provided NMR with an unprecedented level of resolving power and structural specificity. For the first time, complex organic molecules previously intractable by 1D NMR alone could be analyzed systematically and reproducibly. By the late 1980s, 2D NMR had evolved from a groundbreaking innovation into a routine and essential component of every modern chemistry laboratory, firmly establishing itself as one of the most important methodological advances in the history of molecular spectroscopy.

### 2.4. Extension to Biomolecules and Solids

While early applications centered on small molecules, NMR’s scope soon expanded. In biomolecular systems, multidimensional heteronuclear NMR—often enabled by isotopic enrichment with ^13^C and ^15^N—allowed the solution structures of proteins and nucleic acids to be determined with atomic resolution. This represented a paradigm shift: NMR was no longer restricted to connectivity but had become a method for three-dimensional structure determination.

Simultaneously, the development of solid-state NMR opened opportunities to study materials inaccessible to diffraction. Techniques such as magic-angle spinning (MAS) and cross-polarization (CP) enabled relatively high-resolution spectra from powdered samples, leading to applications in polymers, membranes, and inorganic materials, among others.

### 2.5. Establishing NMR as a Cornerstone

By the end of the 20th century, NMR had established itself as a cornerstone technique for structural elucidation across chemistry and biology, with many experiments available [14]. Its strengths lay in: (i) direct connectivity information, (ii) distance and stereochemical constraints (via NOEs, coupling constants, and anisotropic NMR parameters such as residual dipolar couplings, RDCs), (iii) applicability to both ordered and disordered systems, and (iv) ability to probe not only static structures but also dynamics over a wide range of timescales. These foundational contributions laid the groundwork for the subsequent methodological and technological innovations that would continue to expand NMR’s structural reach.

## 3. Advances in Instrumentation and Sensitivity

### 3.1. Growth of Magnetic Field Strength

The evolution of NMR instrumentation has been closely tied to the development of superconducting magnets. The first commercial spectrometers operated at fields of 60–100 MHz for protons, sufficient for rudimentary 1D experiments but inadequate for complex spectra. Over the decades, the achievable field strengths have risen steadily [15], with proton frequencies surpassing 1 GHz in the early 21st century. The impact of increased magnetic field strength on structural elucidation is twofold. First, sensitivity improves with field because the equilibrium magnetization—and thus the Boltzmann population difference between spin states—scales approximately linearly with the static magnetic field under thermal equilibrium conditions. This fundamental relationship underpins the widely accepted high-field approximation that the signal-to-noise ratio (SNR) follows an SNR ∝ B_0_^3/2^ dependence [16]. In practice, however, the achievable SNR may deviate from this ideal behavior due to additional influences such as noise characteristics, relaxation dynamics, and hardware limitations. Second, resolution is dramatically enhanced, since chemical shift dispersion scales directly with magnetic field. For crowded spectra—such as those of complex natural products or proteins—this enhanced dispersion is critical for resolving overlapping resonances. Recent breakthroughs in magnet technology, including the integration of high-temperature superconductors (HTSs), are paving the way toward fields beyond 1.2 GHz. These ultrahigh-field instruments promise not only sharper spectra but also new opportunities to exploit anisotropic interactions, which scale with field strength and become more informative at higher fields.

In addition to the pursuit of ever higher magnetic fields, the advent of cryogen-free superconducting magnet systems represents a major practical and strategic advance for the field. By eliminating the need for liquid helium refills and greatly reducing dependence on cryogen logistics, these systems significantly lower operational costs, simplify maintenance, and improve long-term sustainability. This is particularly relevant in the context of recurring global helium shortages and increasing infrastructure constraints. Moreover, modern cryogen-free platforms have demonstrated excellent field stability and performance suitable for high-resolution applications, making them not merely a logistical improvement but a robust alternative to conventional cryogenic magnets [17].

### 3.2. Pulse Field Gradients

The introduction of pulsed-field gradients (PFGs) in the 1990s represented a major turning point in modern NMR methodology, profoundly enhancing both spectral quality and experimental efficiency. By enabling selective coherence pathway filtering and efficient suppression of undesired magnetization, PFGs greatly reduced the need for extensive phase cycling, thereby shortening acquisition times and improving robustness in routine and multidimensional experiments. Their impact spans from producing ultra-clean spectra in solution-state applications to enabling advanced techniques such as diffusion measurements and solvent suppression, fundamentally reshaping how chemists approach NMR experiment design [18].

### 3.3. Cryogenic Probe Technology

**Figure 4:**
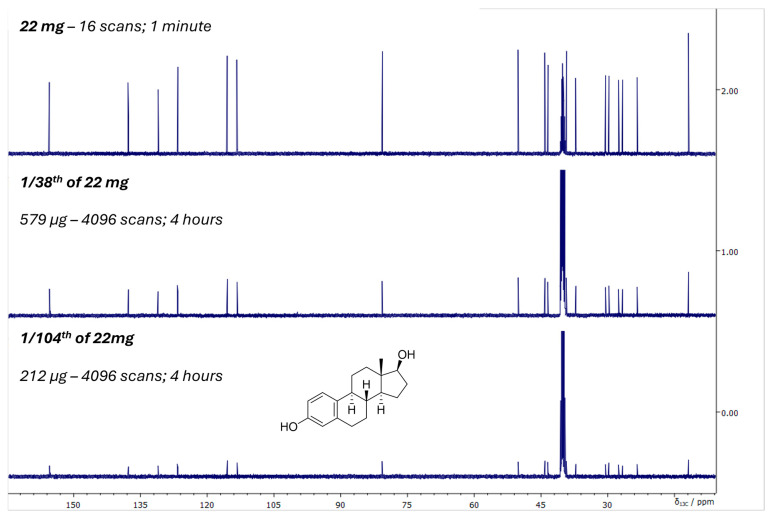
^13^C{^1^H} NMR sensitivity of estradiol acquired on a JEOL magnet equipped with a nitrogen cryoprobe. Spectra are shown for three sample amounts of estradiol: 22 mg (16 scans; 1 min acquisition), 579 µg (1/38 of 22 mg; 4096 scans; 4 h acquisition), and 212 µg (1/104 of 22 mg; 4096 scans; 4 h acquisition). All experiments were recorded with a 90° pulse, 1.4 s acquisition time, and 3.4 s repetition delay. The data illustrate the probe’s ability to detect sub-milligram quantities, achieving approximately 1 mg sensitivity in ~15 min while preserving spectral resolution across more than two orders of magnitude in sample mass.

A third major advance in instrumentation was the development of cryogenically cooled detection systems, or cryoprobes [19]. In conventional probes, electronic noise from room-temperature detection coils and preamplifiers limits sensitivity. By cooling these components to cryogenic temperatures, thermal noise is reduced dramatically, leading to sensitivity enhancements compared to room-temperature probes. Two principal technological approaches have emerged in this context: helium-cooled and nitrogen-cooled cryoprobes. Helium cryoprobes, which cool the detection coil and preamplifier to 20–30 K, deliver the largest sensitivity enhancements, typically providing 3- to 4-fold gains over conventional room-temperature probes. This level of performance has made helium-cooled cryoprobes the gold standard for high-sensitivity biomolecular and small-molecule NMR applications. Their advantages, however, come at the cost of more complex cryogenic hardware and significantly higher operational and maintenance demands. By comparison, nitrogen cryoprobes, operating at 80–90 K, offer more moderate but still substantial sensitivity improvements—generally 2- to 3-fold—reflecting the higher operating temperature of the cooled electronics. While helium cryoprobes remain unmatched in absolute sensitivity, nitrogen-based systems have gained popularity and they offer a compelling compromise between performance, cost, and logistical robustness, broadening access to cryogenic detection technology across a wider range of laboratories. The consequences of having access to cryoprobe technology for structural studies are profound, enabling the acquisition of high-quality spectra from much smaller sample amounts, reducing the typical requirement from tens of milligrams to the low-milligram or even sub-milligram scale (Figure 4). This has made NMR accessible for precious natural products, limited biological samples, and metabolomics studies where material is inherently scarce. Specialized cryoprobes optimized for ^13^C or for multinuclear detection further broaden the applicability of the technology, ensuring that cryogenic enhancement is not restricted to proton-detected experiments alone.

### 3.4. Hyperpolarization Strategies

**Figure 5:**
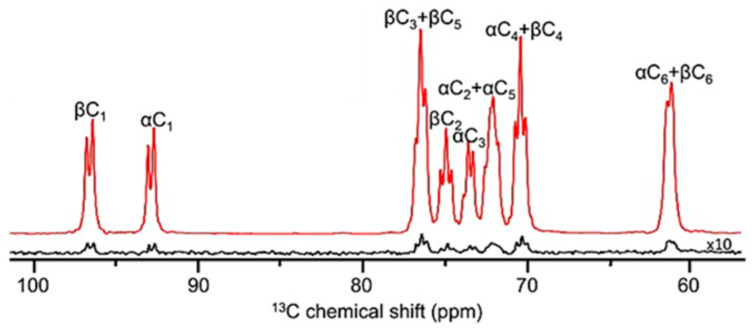
Dissolution dynamic nuclear polarization (DNP) of hyperpolarized U-^13^C,^2^H_7_-glucose showing signal enhancement of ~13,000 times (~13% polarization) observed for a 6.7 mM solution of U-^13^C,^2^H_7_-glucose hyperpolarized after 45 min of microwave irradiation (top trace, red) compared with a thermal equilibrium NMR spectrum (bottom trace, black). Both traces were measured at 125.74 MHz with ^2^H decoupling, the hyperpolarized spectrum by a single scan with a 9° pulse, and the thermal equilibrium spectrum by 16 scans acquired with 45° pulses and a 60 s relaxation delay to ensure full return to thermal equilibrium. Adapted from T. Harris et al. NMR Biomed. 2013; 26: 1831–1843, DOI: 10.1002/nbm.3024. Copyright © 2013 John Wiley & Sons, Ltd.

Despite advances in magnet and probe technology, sensitivity remains a fundamental limitation of NMR, arising from the inherently small nuclear spin polarization at thermal equilibrium. To overcome this, hyperpolarization techniques aim to artificially enhance nuclear polarization by several orders of magnitude [20]. The most widely developed approach is Dynamic Nuclear Polarization (DNP), in which polarization is transferred from unpaired electrons (with much higher magnetic moments) to nuclei under microwave irradiation [21,22], providing an astonishing enhancement of the nuclear polarization (Figure 5). Other hyperpolarization strategies include parahydrogen-induced polarization (PHIP) [23] and signal amplification by reversible exchange (SABRE) [24], which exploit the spin order of parahydrogen to enhance signals in solution-state NMR. These approaches are increasingly being applied in solution and solid state.

### 3.5. Miniaturization and Benchtop Instruments

In parallel with the drive toward ever larger magnets, a complementary trend has emerged toward miniaturization and portability. Permanent-magnet-based benchtop NMR spectrometers operating at fields of 60–100 MHz have become commercially available and are finding applications in quality control, education, and reaction monitoring and metabolomics [25]. While their resolution and sensitivity are limited compared to high-field instruments, their accessibility and robustness make them valuable tools for routine structural tasks outside of specialized facilities.

More recent efforts focus on benchtop-compatible DNP equipment. Recent instrument-development efforts are producing compact DNP polarizers designed to operate adjacent to small NMR magnets [26]. As these benchtop DNP platforms mature, they promise to make high-sensitivity, multi-scan hyperpolarized spectroscopy feasible without the infrastructure of high-field magnets or cryogenic equipment.

Microcoil and capillary probes represent another path toward miniaturization, focusing on reducing detection volume to the microliter scale. By increasing the filling factor and reducing noise, such probes enable the analysis of tiny samples, including single cells or microscale reaction mixtures [27].

### 3.6. Impact on Structural Elucidation

Taken together, these instrumental advances have transformed the practice of NMR. Where once spectra required milligram-to-gram quantities of analyte and lengthy acquisition times, today’s high-field, cryogenically enhanced instruments can deliver multidimensional datasets from microgram quantities in hours or less. Hyperpolarization has opened new frontiers by making previously undetectable species observable. Benchtop systems are democratizing access to NMR, bringing it into laboratories that would not otherwise have access to such technology.

For structural elucidation, the practical outcome is clear: NMR is now applicable to a wider range of samples, from scarce natural products to complex biomolecular assemblies, and can reveal structural information with unprecedented sensitivity and resolution.

## 4. Methodological Developments

### 4.1. Multidimensional NMR

While instrumentation advances provided improvements in sensitivity and resolution, the methodological innovations of NMR spectroscopy have been equally transformative. The extension from 1D to 2D experiments in the 1970s and 1980s was only the beginning; today, multidimensional NMR experiments are routine.

For small molecules, 2D experiments such as HSQC, COSY and HMBC remain the workhorses of structure elucidation, but an increasingly diverse suite of correlation techniques has emerged over the years to address specific challenges in resonance assignment and connectivity analysis. TOCSY (Total Correlation Spectroscopy), which was already developed in the early 1980s [28], extends the capabilities of COSY by relaying magnetization across entire scalar-coupled spin networks, making it indispensable for identifying complete spin systems in densely substituted molecules or complex mixtures. Expanding upon this, HSQC-TOCSY incorporates the sensitivity and heteronuclear resolution of HSQC with the spin-system mapping power of TOCSY [29], enabling long-range proton networks to be traced through ^13^C-edited correlations, especially useful when proton spectra alone exhibit severe overlap [30].

**Figure 6:**
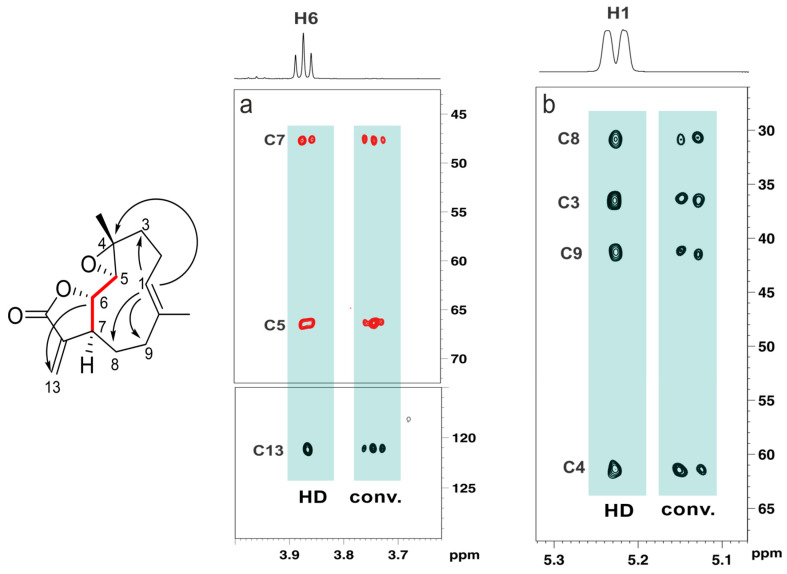
Expansion of the 2D dual-optimized inverted ^1^*J*CC 1,n-HD-ADEQUATE spectra of parthenolide, showing correlations originating from (**a**) proton H6 and (**b**) proton H1. The experiment enables editing of ^1^*J*CC and ^n^*J*CC responses, while also providing enhanced sensitivity through real-time BIRD-based homodecoupling. ^n^*J*CC correlations are shown in black (and indicated by black arrows in the structural drawing), while ^1^*J*CC correlations are shown in red (and indicated by red bonds in the structural drawing). For clarity, responses from the conventional, non-HD experiment are slightly shifted to the right.

The dominance of heteronuclear inverse-detection experiments (HSQC/HMBC) is closely linked to their better sensitivity compared to direct methods [31]. However, this advantage diminishes in proton-deficient molecules where the lack of attached protons significantly reduces polarization transfer efficiency. This limitation explains the rise of a modern broad suite of complementary experiments targeting specific connectivity pathways that are otherwise difficult to access. ADEQUATE (Adequate Double-Quantum Transfer Experiment) [32] provides unambiguous identification of two-bond carbon–carbon connectivities through ^13^C-^13^C double-quantum coherences. Although inherently constrained by the low natural abundance of ^13^C, the 1,1-ADEQUATE experiment—along with several of its modern variants [33]—remains one of the few experiments capable of delivering direct skeletal connectivity maps, particularly advantageous in structures containing ambiguous carbon frameworks. Importantly, the adoption of cryogenically cooled probes significantly mitigates the sensitivity limitations of these experiments, enabling the acquisition of high-quality ADEQUATE spectra even for samples available only in limited quantities, thereby expanding the practical applicability of these connectivity-based experiments to more challenging structural elucidation problems. For instance, a state-of-the-art experiment for structure elucidation purposes is the dual-optimized, inverted ^1^*J*CC 1,n-ADEQUATE experiment incorporating real-time BIRD-based homodecoupling (HD). The experiment yields broadband inversion of direct ^1^*J*CC responses with respect to ^n^*J*CC responses, obviating the need to take additional steps to distinguish these correlations and remarkably facilitating the unambiguous assignment of a carbon skeleton, while the incorporation of HD acquisition further improves sensitivity by partly collapsing homonuclear proton–proton couplings, simplifying multiplet structures and enhancing spectral clarity. As a result, the method greatly facilitates the unambiguous assignment of a molecule’s carbon skeleton while affording superior sensitivity compared to the conventional pulse sequence (Figure 6). Complementing this, long-range heteronuclear experiments such as LR-HSQMBC (Long-Range Heteronuclear Single Quantum Multiple Bond) enhance the detection of weak long-range C–H couplings [34], significantly improving spectral clarity for quaternary centers, remote substituents, and extended fragments where traditional HMBC correlations are insufficient.

Selective pulse strategies, a concept already explored in early years of NMR, have also become central to modern small-molecule NMR. Selective TOCSY, selective NOESY/ROESY and other tailored experiments allow for targeted interrogation of specific resonances or functional groups, reducing spectral crowding and enabling precise disambiguation of overlapping signals. These approaches are particularly powerful in mixture analysis or conformational studies and offer a rapid way to access valuable information. Together, these expanded methodologies complement and extend the traditional HSQC–COSY–HMBC triad, providing a more comprehensive, versatile, and reliable toolkit for small-molecule structural elucidation across increasingly complex chemical space.

In biomolecular NMR, the development of 3D and 4D correlation experiments has provided powerful means to resolve ambiguities in crowded spectra. Multidimensionality is indispensable in this field where triple-resonance experiments involving ^1^H, ^13^C, and ^15^N nuclei are the backbone of protein assignment strategies, enabling residue-by-residue mapping of large macromolecules.

Equally important has been the extension of correlation strategies to other nuclei. Amongst these, ^19^F NMR is widely considered one of the most powerful and versatile nuclei in modern NMR spectroscopy. Its importance comes from a combination of unique nuclear properties, high sensitivity, and broad applicability across chemistry, materials science, and drug discovery [35].

### 4.2. Pulse-Sequence Development

Pulse-sequence development has been a foundational element of NMR research since its inception, and it continues to be one of the most dynamic areas of innovation in the field. In recent years, substantial advances in pulse-sequence design have been driven by the persistent goals of improving spectral resolution, enhancing sensitivity, increasing selectivity and expanding the range of structural information accessible from small-molecule samples. Although a comprehensive treatment of these developments lies beyond the scope of this manuscript, it is worth highlighting some aspects in the ongoing progress in this arena, which continues to influence and reshape current strategies for NMR-based structure elucidation. These innovations impact both routine and advanced workflows, underscoring the central role of pulse-sequence engineering in modern NMR spectroscopy.

Homonuclear decoupling continues to evolve from its foundational role in spectral simplification toward increasingly specialized and application-driven implementations. Much progress has been made in the pure-shift methodological area in the last 15 years, with advances ranging from solvent-suppressed pure-shift sequences [36] to pure-shift diffusion experiments [37], continuous-flow-compatible pure-shift acquisitions [38], and new schemes for extracting homo- and heteronuclear coupling constants [39].

Developments in multi-experiment acquisition strategies have also been noteworthy. Multiple-FID acquisition techniques have been implemented aiming at collecting several free induction decays within a single recycle delay, typically following one excitation or preparation period. This is achieved by interleaving acquisition blocks with brief delays or refocusing elements, allowing the spectrometer to sample signal evolution more efficiently without full relaxation between scans. The primary goal of Multiple-FID acquisition is to improve experimental efficiency by increasing sensitivity per unit time and reducing overall acquisition time, particularly in experiments limited by long relaxation delays [40]. NOAH supersequences [41], which nest multiple 2D modules within a single relaxation delay, have also been recently introduced and they continue to expand in scope and flexibility. Recent implementations include automated code generation, magnetization-recycling schemes, and vertically interleaved designs that pair long, low-sensitivity modules (such as 1,1-ADEQUATE) with rotating sets of more sensitive modules (e.g., HSQC, HMBC, NOESY). Isotope-selective elements, including tailored zz-type filters, preserve proton magnetization attached to 12C for subsequent modules, allowing for efficient balancing of sensitivity across the supersequence. Five-module variants that combine heteronuclear, homonuclear, and NOE-based experiments illustrate how NOAH strategies can deliver comprehensive structural information with a fraction of the traditional acquisition time, without significantly compromising spectral quality [42].

Pulse sequences aimed at more accurate extraction of scalar couplings have also diversified, becoming sophisticated at times, yet very elegant and powerful methods. These include pure-shift approaches [39], 2D *J-*resolved methods [43,44], or spin-state selective methods [45,46,47]. The development of new tools to improve the quality of NMR spectra using the concept of “perfect NMR”, which yields undistorted pure in-phase signals with pure absorption lineshapes, has also found many applications [48]. Among the newest developments, the recently introduced i-HMBC experiment provides a sensitive and universal strategy to unequivocally identify two-bond heteronuclear correlations by exploiting subtle isotope-induced chemical-shift changes in HMBC spectra [49], a feature with huge potential impact on structure elucidation protocols.

A central line of progress stems from the GEMSTONE concept [50], originally developed to achieve ultra-selective excitation in situations of severe spectral overlap. Its core principle—combining chemical-shift filtering with spatial encoding—has now been adopted as a versatile building block for multiple selective 1D correlation experiments.

Finally, diffusion-based pulse sequences have also continued to evolve, expanding the analytical capabilities of DOSY-type methods [51].

### 4.3. Non-Uniform Sampling and Fast Acquisition Strategies

Traditional multidimensional NMR experiments rely on uniform sampling of indirect time domains, resulting in acquisition times that scale exponentially with dimensionality. For high-resolution 3D or 4D datasets, this quickly becomes prohibitive. Non-uniform sampling (NUS) strategies [52] overcome this limitation by acquiring only a subset of the data points and reconstructing the full spectrum using advanced algorithms such as maximum entropy, compressed sensing, or iterative soft thresholding. The adoption of NUS has revolutionized multidimensional NMR, making it possible to acquire high-resolution 3D protein spectra in hours instead of days, or to collect multiple 2D spectra in rapid succession for kinetics or metabolomics studies. In small-molecule NMR, NUS enables high-throughput acquisition of multidimensional datasets, significantly accelerating structure elucidation workflows [53].

Complementary developments include ultrafast NMR, in which entire multidimensional datasets are encoded within a single scan using spatial encoding and gradient technology [54]. Although technically demanding, ultrafast methods hold promise for real-time reaction monitoring and dynamic studies [55].

### 4.4. Anisotropic NMR Parameters

**Figure 7:**
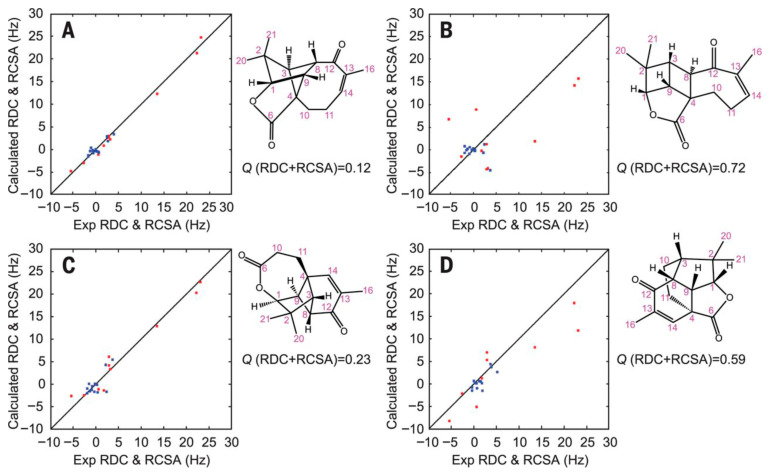
Plots of the calculated versus experimental RDC (red) and RCSA (blue) data for aquatolide candidate structures. (**A**) The revised structure; (**B**) the originally proposed incorrect structure of aquatolide; and (**C**,**D**) two alternative structures. Clearly there is a vast difference in the Q value for the correct structure and the originally reported structure of aquatolide. The alternative structures generated by CASE had intermediary Q values of 0.23 and 0.59, respectively. A twofold difference in the Q value between the correct structure and the best alternative structure from the CASE program still allowed an ample basis for choosing between the structures. Reproduced from ref. [57], Yizhou Liu et al., Science, DOI: 10.1126/science.aam5349 (2017), AAAS.

Traditional solution-state NMR exploits isotropic tumbling, which averages out anisotropic interactions such as residual dipolar couplings (RDCs), residual chemical shift anisotropy (RCSA) and residual quadrupolar couplings (RQCs). However, partial alignment of molecules reintroduces anisotropic terms that can be exploited for structural purposes. Although these parameters were initially introduced to help tackle protein assignment strategies, they have had a significant impact on modern structure-elucidation protocols of small- to medium-size molecules by providing long-range, orientation-dependent information that complements traditional isotropic observables. Unlike scalar couplings and NOEs, anisotropic parameters report on the relative orientation of internuclear vectors and molecular fragments with respect to a common alignment frame, enabling discrimination between closely related stereochemical models and conformations. The typical workflow follows a combined computational–experimental strategy to evaluate candidate molecular structures using DFT and anisotropic NMR parameters. Putative stereoisomers are first generated and subjected to a conformational search. Representative conformers are then optimized at the DFT level, including vibrational frequency calculations to confirm true minima, followed by GIAO-DFT computation of chemical shielding tensors. The resulting low-energy conformers are used for RDC and RCSA back-calculation, and experimental RDC/RCSA data are incorporated through SVD-based structural analysis. Final structure selection is achieved by comparing Q-factors, allowing for reliable discrimination between correct and incorrect stereochemical assignments. The incorporation of anisotropic NMR parameters into structure-validation and refinement workflows has greatly increased the reliability of configurational assignments, particularly for semi-flexible molecules and complex natural products (Figure 7), and has helped resolve ambiguities that are otherwise inaccessible using isotropic NMR data alone [56,57]. Some of the most recent research using anisotropic NMR parameters for structure elucidation purposes have found applications in determining the absolute configuration of natural products [58], even flexible ones [59].

Many methods for generating the required weak molecular alignment in solution have been developed over the years, and recent work continues to expand the range of available anisotropic media and approaches. Recent examples include peptide-based lyotropic liquid crystals [60,61], chiral polymer-based alignment phases [62,63], helically chiral polyarylacetylene systems [64], self-assembled oligopeptides [65], polymer-gel alignment media [66], and biological filament-based media [67]. These studies represent some of the latest developments in solution-state anisotropic NMR methodologies.

### 4.5. Solid-State NMR Advancements

Solid-state NMR has undergone dramatic methodological progress in recent decades, transforming from a niche technique into a mainstream structural tool [68]. The advent of magic-angle spinning (MAS) at ever higher spinning frequencies—now exceeding 150 kHz—has enabled near-liquid-like resolution for protons in solids [69]. Techniques such as Cross-Polarization (CP), dipolar recoupling, and Rotational-Echo Double-Resonance (REDOR) permit the measurement of internuclear distances and connectivities, providing detailed structural information even in non-crystalline materials [70]. Determining the structure of large biomolecules, particularly proteins, has become increasingly widespread in solid-state NMR research. Among the available methodologies, Dipolar Assisted Rotational Resonance (DARR) is a commonly employed technique for studying such systems in the solid phase. In ^13^C-enriched samples, DARR experiments provide ^13^C–^13^C correlation spectra, enabling the characterization of both intra- and intermolecular structural features. Last but not least, DNP, when coupled with MAS, has extended sensitivity by orders of magnitude, making it possible to study interfaces, surfaces, and low-concentration species in complex matrices [71].

Applications span from the structural characterization of pharmaceutical polymorphs to the study of amyloid fibrils, battery materials, and heterogeneous catalysts. Importantly, solid-state methods bridge a critical gap by allowing for the structural investigation of systems inaccessible to diffraction or solution-state techniques.

### 4.6. Impact on Structure Elucidation

Collectively, these methodological innovations have redefined the practice of NMR. Novel pulse sequences and NUS strategies have increased spectral clarity and acquisition efficiency, while anisotropic methods provide information inaccessible to classical approaches. Solid-state NMR has opened vast new areas of application, ensuring that structural elucidation is not limited to molecules soluble in favorable solvents. The outcome is an NMR toolbox of extraordinary versatility: whether the challenge is resolving crowded spectra, probing flexible biomolecules, or studying amorphous materials, modern NMR methodologies offer tailored solutions. These advances have set the stage for deeper integration with computation, a topic explored in the following section.

## 5. Computational Synergies

### 5.1. From Manual Interpretation to Computational Assistance

For much of its history, NMR-based structure elucidation depended on the expertise of trained spectroscopists, who interpreted chemical shifts, coupling patterns, and correlations directly from experimental spectra. This hands-on practice fostered a deep, intuitive understanding of molecular behavior and laid the foundations of modern NMR analysis. As molecular targets became more complex and datasets more information-rich, these interpretive methods evolved naturally toward increasingly automated, assisted, and computationally supported approaches that build upon (rather than replace) the original conceptual framework.

Early structural analysis relied largely on qualitative pattern recognition, with characteristic motifs inferred from chemical shifts and coupling signatures. As applications extended to more subtle structural problems, interpretation required progressively quantitative models, including calculated chemical shifts and coupling constants, NOE-derived distance restraints, and later anisotropic observables such as RDCs. This progression reflects a broader trend: contemporary and emerging challenges increasingly hinge on detecting and interpreting very small NMR perturbations, often in regimes where empirical pattern libraries are sparse or unavailable—particularly in RDC-based analyses of complex small molecules. Advances in computing, quantum chemistry, and algorithmic analysis have transformed the field. Experiment and theory have not only become individually more sophisticated, but are now tightly intertwined in an iterative dialog with nature, in which computational predictions can guide experimental design, and experimental results, in turn, refine theoretical models. Computation thus acts as both a complement to and an extension of experiments, supporting all stages of NMR analysis from spectral assignment to complete structure determination.

### 5.2. Quantum Chemical Calculations of NMR Parameters

Among the most powerful computational tools for NMR are quantum chemical calculations of chemical shifts, spin–spin couplings, and other observables. Early semiempirical methods provided only rough semiquantitative estimates, but the introduction of Density Functional Theory (DFT) in the 1990s made accurate and affordable calculations possible for molecules of realistic size.

DFT-based predictions of chemical shifts have since become an integral part of structural elucidation workflows [4]. By comparing experimental and calculated chemical shifts for candidate structures, chemists can assess which structure or configuration best matches the data. This approach has proved especially valuable in the determination of relative stereochemistry in complex natural products, where subtle differences in shielding patterns can distinguish between diastereomers. Spin–spin coupling constants can also be computed at high accuracy, offering complementary information about dihedral angles and bonding pathways. When combined with experimental data, such calculations facilitate not only validation but also refinement of proposed structures.

In this context, the DP4 probability analysis, later refined into DP4+ and related Bayesian frameworks, formalized this comparison by quantifying the likelihood that a given structure corresponds to the observed data. These methods have been validated across hundreds of examples and are now standard tools for structure validation [72]. Although often described as probabilistic, chemical-shift-based structure validation methods such as DP4/DP4+ are fundamentally Bayesian: the predicted shifts are deterministic DFT values, but the analysis evaluates the likelihood of each candidate structure given assumed error models. The author of this manuscript argues that although DP4-based methodologies have become the prevailing tools for the stereochemical assignment, a number of limitations that can mislead the analysis persist, including (i) overconfident posterior probabilities sensitive to the choice of structural candidates, (ii) sensitivity to conformer sampling, particularly for structurally complex systems where conformational sampling is incomplete or uncertain, (iii) assumptions of independent DFT prediction errors and (iv) known DFT failure modes—solvent effects [73] and heavy-atom environments.

As described in previous sections, DFT calculations also play a central role in the determination and interpretation of anisotropic NMR parameters. By transforming experimentally measured observables into quantitative descriptors of the underlying electronic structure and molecular geometry, DFT provides the essential link between NMR data and structural models [56].

### 5.3. Computer-Assisted Structure Elucidation (CASE) Methods

A parallel line of development has focused on computer-assisted structure elucidation (CASE), which aims to automate the interpretation of NMR data into candidate molecular structures. Early systems such as CSEARCH and LUCY relied on heuristic rules and pattern recognition to match spectra against structural databases. Later generations, including ACD/Structure Elucidator and SESAMI, incorporated more sophisticated logic-based algorithms and probabilistic reasoning. Modern CASE systems integrate multiple data sources—^1^H, ^13^C, and 2D correlation spectra—to generate all structures consistent with the data [74]. These are then ranked according to consistency metrics, including chemical shift predictions from quantum chemistry or machine learning. In many cases, the correct structure is identified automatically, reducing the need for manual interpretation.

Beyond small molecules, similar computational logic underpins automated workflows in biomolecular NMR, where resonance assignment and structure calculation are handled by software such as CYANA, ARIA, or XPLOR-NIH. Although these tools rely heavily on distance and coupling restraints rather than direct chemical reasoning, the conceptual similarity, i.e., converting spectral data into structural models, is unmistakable.

### 5.4. Integration of Experiment and Computation

The true power of computation in NMR emerges not merely from individual tools, but from their integration into cohesive workflows. In contemporary practice, experimental spectra and calculated parameters are used iteratively: initial spectral assignments guide structural models, which are then validated and refined by comparison with quantum chemical predictions. For example, iterative DFT–NMR refinement has become a standard protocol for natural product structure verification [59]. The process begins with experimental data suggesting one or more candidate structures. DFT calculations are performed for each, yielding predicted chemical shifts and coupling constants. Statistical comparison with the experimental dataset allows for the rejection of incorrect structures and, in some cases, the correction of published misassignments.

Integration is further facilitated by machine-readable data formats (such as NMReDATA and JCAMP-DX) and open spectral repositories (like nmrshiftdb2), which make it possible to link raw experimental data with computational analyses in a reproducible manner.

### 5.5. Machine Learning Prediction

In recent years, machine learning (ML) has emerged as a powerful alternative to traditional quantum chemical prediction. ML models trained on large databases of experimental spectra can predict chemical shifts and coupling constants with remarkable accuracy and at negligible computational cost. Techniques ranging from kernel methods and random forests to deep neural networks have been applied successfully [75]. A particularly exciting development is the use of graph neural networks (GNNs) that operate directly on molecular structures, learning to predict NMR parameters from atom connectivity and local environments. Some models now approach or even surpass DFT-level accuracy for certain nuclei, enabling near-instantaneous prediction and comparison [76,77].

ML is also being integrated into automated spectral analysis, where algorithms can deconvolute complex spectra, identify peak patterns, and suggest structural fragments [78,79]. In combination with CASE and DFT-based validation, such approaches are expected to provide fully automated structure elucidation pipelines that can handle large-scale datasets—such as those encountered in metabolomics or reaction screening.

### 5.6. Outlook

The synergy between computation and experiment has fundamentally reshaped NMR structural elucidation. Quantum chemistry provides physical grounding and interpretive power; CASE systems automate reasoning; and machine learning offers unprecedented speed and scalability. Together, they are blurring the boundary between measurement and modeling, creating a hybrid discipline in which computation is as indispensable as the spectrometer itself. As these tools continue to mature, the future of NMR will increasingly depend on seamless human–machine collaboration—where experimental insight and algorithmic analysis converge to accelerate discovery.

## 6. Expanding Applications

**Figure 8:**
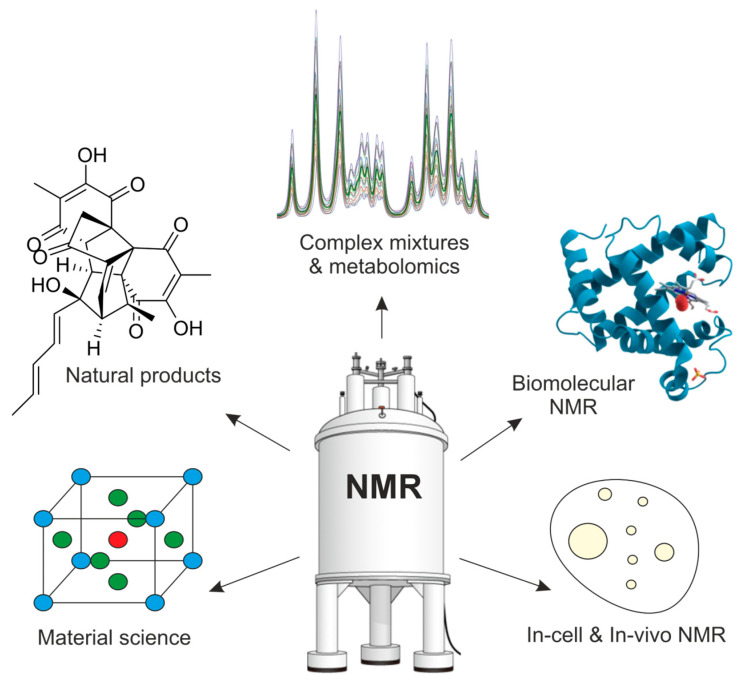
Expanding application landscape of modern NMR spectroscopy. Advances in NMR have broadened its impact across diverse fields, including natural product structure elucidation, complex mixture analysis and metabolomics, biomolecular NMR, materials science, and in-cell and in vivo studies.

The continuing evolution of NMR methodology, instrumentation, and computational integration has dramatically broadened the scope of its applications. What began primarily as a tool for molecular structure determination has expanded into a versatile platform capable of addressing increasingly complex problems, ranging from scarce natural products and multicomponent mixtures to living cells, functional materials, and intact organisms (Figure 8).

### 6.1. Natural Products

NMR spectroscopy has always played a central role in the structural elucidation of natural products, a field that continues to test the limits of spectroscopic methods. Natural products often feature high molecular complexity—either dense proton or proton-deficient environments—multiple stereocenters, and flexible conformations that challenge even the most experienced spectroscopists. As described in previous sections, over the past two decades, methodological and computational progress has significantly extended the reach of NMR in this area. Multidimensional correlation experiments, especially HSQC, COSY, HMBC and NOESY, remain indispensable tools for constructing the molecular framework, yet more sophisticated experiments are being introduced in the toolkit. The use of high-field instruments and cryogenic probes has dramatically improved signal-to-noise ratios, allowing the elucidation of structures from sub-milligram quantities of compound. When coupled with non-uniform sampling (NUS) and state-of-the-art pulse sequences, even tiny amounts of material can yield complete datasets suitable for full structure determination. At the same time, the combination of experimental data with quantum chemical shift calculations and CASE methods has become a standard practice in natural product chemistry and they are now routinely employed to confirm or correct proposed structures. Numerous cases exist where initial NMR-based assignments were later revised through computational validation, underscoring the growing synergy between theory and experiment [80]. Despite progress, several major challenges remain in natural product elucidation: proton-deficient frameworks, flexible polycyclic systems with broad conformational manifolds, and molecules requiring unambiguous C–C connectivity. A major step-change could come from either i) sufficient sensitivity to perform routine INADEQUATE or 1,1-ADEQUATE experiments on microgram samples, or ii) chemical-shift predictions approaching ~0.02 ppm accuracy, which would dramatically reduce the need for extensive experimental suites.

### 6.2. Complex Mixtures

The inherently non-destructive character of NMR makes it particularly well-suited for investigating complex mixtures without requiring prior separation. This capability is reinforced by a continually expanding toolkit of experimental methods and pulse sequences specifically designed to address the challenges presented by small-molecule mixtures, such as overlapping signals, low-abundance components, and dynamic composition. Recent developments—including pure-shift techniques, hyperpolarization approaches, diffusion-based NMR, and accelerated multidimensional methods like ultrafast 2D NMR and non-uniform sampling—have significantly broadened the scope of accessible studies. These approaches are increasingly employed across a range of applications, underscoring both the versatility of NMR for deconvoluting complex systems and the importance of selecting the most appropriate methodology for a given analytical problem [81].

**Figure 9:**
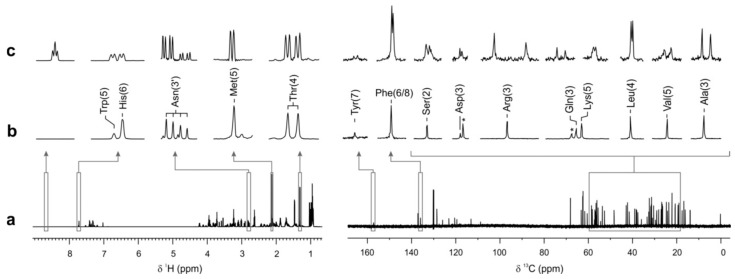
Simultaneous enantiospecific detection of an amino acids (AA) mixture by NMR spectroscopy. (**a**) 1D ^1^H and ^13^C NMR spectra of mixture containing the nineteen chiral essential AAs present as racemates plus glycine. (**b**) Highlighted ^1^H and ^13^C NMR signals of the same mixture. (**c**) Same NMR signals after the addition of 3.1 mg (final concentration 11.7 mM) CSA (−)-18C6H4, which induced enantiospecific changes in the spectrum that were sufficient to enantiodifferentiate most of the enantiomeric pairs of AAs in the mixture. Adapted from ref. [82], Lars T. Kuhn et al. Angew. Chem. Int. Ed. 2020, 59, 23615–23619. DOI: 10.1002/anie.202011727.

A remarkable development for mixture analysis has been the emergence of simultaneous enantiospecific detection within multicomponent samples—a longstanding challenge in chiral analysis. Traditionally, NMR-based enantiomeric studies require prior chromatographic separation or the individual derivatization of each component, making enantioselective profiling of complex mixtures impractical. Recent work [82] provided proof-of-concept that enantiomer-level molecular information can be extracted directly from highly complex mixtures without any prior separation, opening the door toward multicomponent enantiospecific metabolite analysis under native-like conditions with implications for metabolism studies, metabolic phenotyping, and chemical reaction monitoring, among others (Figure 9).

### 6.3. Metabolomics

The rise of metabolomics has further broadened NMR’s role in the study of complex systems. In contrast to targeted natural product work, metabolomics emphasizes high-throughput, quantitative, and reproducible profiling of small-molecule metabolites across biological samples. Here, NMR’s quantitative accuracy and reproducibility—stemming from its linear response and minimal sample preparation—offer key advantages over mass spectrometry. ^1^H NMR spectra can provide holistic fingerprints of biofluids, tissues, or cell extracts, which, when analyzed statistically, reveal patterns associated with disease states, environmental changes, or genetic perturbations [83].

Automated data processing pipelines, including bucketing, alignment, and multivariate analysis (PCA, PLS-DA), are now routine. More recently, the integration of NMR with computational metabolite databases (e.g., HMDB, BMRB, and nmrshiftdb2) and machine learning-based spectral matchers have accelerated compound identification. Emerging approaches such as 2D qNMR [84] and hyphenated NMR–MS workflows are pushing toward comprehensive, quantitative metabolomic profiling at unprecedented depth. Ultrafast NMR methods have also emerged as powerful tools for high-throughput metabolite analysis, significantly reducing experiment times and enabling near real-time screening of metabolic profiles [85].

The combination of hyperpolarization strategies with NMR metabolomics has emerged as a powerful strategy to overcome the intrinsic sensitivity limitations of NMR spectroscopy, enhancing NMR signal intensities by several orders of magnitude and hence enabling the detection of low-abundance metabolites while reducing acquisition times [86]. Complementary to these methods, photo-CIDNP provides light-induced hyperpolarization of specific nuclei through spin-selective photochemical reactions, offering selective signal enhancement for metabolites involved in photoactive or radical-pair processes [87]. The integration of DNP and photo-CIDNP with NMR metabolomics thus enables both global sensitivity enhancement and targeted amplification of key molecular species, opening new avenues for quantitative, time-resolved, and pathway-specific metabolic investigations.

### 6.4. Biomolecular NMR

As stated earlier, the introduction of isotopic labeling and multidimensional correlation methods in the 1980s and 1990s established NMR as a third pillar of structural biology, alongside X-ray crystallography and cryo-electron microscopy. Today, NMR enables atomic-resolution characterization of proteins, nucleic acids, and their complexes in solution, providing information not only about static structures but also about dynamics, folding, and interactions. These capabilities are particularly valuable for systems that resist crystallization or display intrinsic disorder.

Developments such as TROSY (Transverse Relaxation-Optimized Spectroscopy) and deuteration strategies have extended the size limit of NMR-accessible proteins to well over 100 kDa, while solid-state NMR allows for structural analysis of fibrils, membrane proteins, and large assemblies under near-native conditions. Residual dipolar couplings, paramagnetic effects, and relaxation dispersion experiments provide insight into long-range order and conformational dynamics across timescales from picoseconds to seconds [88]. NMR’s unique ability to observe conformational ensembles—rather than single static structures—makes it indispensable for understanding intrinsically disordered proteins (IDPs), which play key roles in regulation, signaling, and disease. In such systems, the NMR-derived parameters are combined with computational modeling to describe dynamic energy landscapes inaccessible to crystallographic techniques [89].

### 6.5. Materials Science and Solid-State Applications

In materials research, NMR has evolved from a complementary technique into a central method for elucidating structure and dynamics in solids, amorphous materials, and hybrid systems. Unlike diffraction, which depends on long-range order, solid-state NMR (ssNMR) probes local environments, providing atomic-level insights into both ordered and disordered regions. Applications span a wide range, i.e., pharmaceuticals where ssNMR identifies crystalline forms, polymorphs, and amorphous phases, aiding regulatory and patent analyses [90], catalysis where surface-selective and DNP-enhanced ssNMR reveal the structure of active sites in heterogeneous catalysts [91], energy materials where NMR probes ion dynamics and local environments in batteries, fuel cells, and solid electrolytes [92], and polymers and composites where information on chain conformation, crystallinity, and interfacial interactions is accessible [93].

The combination of high-field magnets, fast MAS, and multidimensional correlation has made it possible to obtain near-solution-quality spectra even for solids. Complementary computational simulations of chemical shielding tensors, often performed at the DFT level, allow for unambiguous assignment of local structural motifs [68].

### 6.6. In-Cell and In Vivo NMR

Beyond isolated molecules and materials, NMR increasingly operates within living systems. In-cell NMR enables the observation of proteins and metabolites directly inside living cells, revealing how molecular structure and dynamics are influenced by the cellular environment. Isotopic labeling, microcoil detection, and advanced pulse sequences have made such experiments feasible even in complex cellular matrices.

In the medical realm, in vivo magnetic resonance spectroscopy (MRS) extends NMR’s structural insights to metabolic processes in intact tissues. While resolution is lower than in vitro spectroscopy, MRS provides non-invasive access to key biochemical pathways in real time, complementing the imaging information obtained from MRI. Hyperpolarization techniques, particularly DNP and parahydrogen-based methods, are expanding the sensitivity of in vivo NMR and enabling dynamic metabolic imaging of living organisms [94].

### 6.7. Toward Integrative Applications

A striking trend across all domains is the integration of NMR with complementary methods. In chemistry, NMR data are combined with mass spectrometry for structure validation and quantification. In structural biology, hybrid approaches merging NMR, cryo-EM, and small-angle scattering allow for the reconstruction of large, flexible assemblies that are beyond the reach of any single technique [95]. In materials science, the combination of NMR with diffraction, microscopy, and computational modeling yields multi-scale structural descriptions that link local and global order [4]. These integrative applications reflect a broader shift: NMR is no longer viewed as an isolated technique but as a component of multi-modal structural analysis, valued for its quantitative, site-specific, and dynamic information.

## 7. Conclusions and Outlook

Since its inception, NMR spectroscopy has been characterized by an extraordinary ability to translate atomic-level observations into molecular understanding. Few analytical techniques rival its versatility: NMR is simultaneously local and global, static and dynamic, qualitative and quantitative. This unique combination has allowed it to serve as both an analytical workhorse and a fundamental research tool across chemistry, biology, and materials science.

Over the past seventy years, the field has evolved from simple 1D proton spectra to sophisticated, multidimensional, and multidomain experiments probing systems of increasing complexity. Each stage of this evolution—Fourier transformation, multidimensional correlation, pulsed-field gradients, high-field magnets, cryogenic probes, solid-state techniques, and, most recently, hyperpolarization—has pushed the boundaries of sensitivity, resolution, and interpretability. At the same time, NMR has continuously reinvented itself conceptually. From the early focus on chemical connectivity, it has expanded toward conformational dynamics, intermolecular interactions, and systems-level behavior. This intellectual adaptability ensures that NMR remains a living and evolving science rather than a mature, static method.

One of the defining features of the current era is the convergence of experimental and computational approaches. The integration of quantum chemical calculations, machine learning, and automation has initiated a shift from interpretive to predictive NMR. Instead of manually assigning peaks and deducing structures, researchers increasingly simulate spectra from candidate models, compare them with experiments, and refine both iteratively. This trend parallels developments in other fields, such as crystallography and cryo-EM, where hybrid modeling and refinement protocols are now standard. For NMR, the outcome is not merely improved structural accuracy but a redefinition of what constitutes “structural information.” Data are no longer treated as static outputs but as dynamic constraints within a computational framework that continuously tests hypotheses against experiments. While automation and AI promise to revolutionize NMR workflows, human insight remains indispensable. NMR spectra are rich in subtle information—small chemical shift perturbations, unexpected cross-peaks, or line-shape changes—that often reveal underlying phenomena such as conformational exchange, aggregation, or binding. Recognizing and interpreting such subtleties requires a conceptual understanding of spin physics, molecular behavior, and experimental limitations that cannot yet be fully captured by algorithms. Moreover, the creative design of experiments and the ability to ask the right questions and devise the pulse sequence remains a fundamentally human endeavor. Automated systems may generate structures, but it is the scientist who formulates the hypothesis, defines the experimental objective, and interprets the results within a broader chemical or biological context.

The future of NMR should therefore not replace the spectroscopist but amplify their capability, allowing them to operate at higher levels of abstraction and productivity. Looking ahead, the evolving landscape of NMR structural elucidation will be defined by integration of methods, modalities, and disciplines. High-field magnets and cryoprobes will continue to enhance sensitivity and resolution, while hyperpolarization methods promise to extend NMR’s reach into previously inaccessible regimes of time and concentration. Non-uniform sampling, dynamic nuclear polarization, and ultrafast multidimensional techniques will further accelerate data acquisition and expand experimental design. Simultaneously, advances in computation will transform how we think about and interact with NMR data. The increasing availability of open repositories, standardized formats, and shared analysis frameworks will enable large-scale data mining and model training. Coupled with developments in AI and cloud-based computation, this will lead to an ecosystem in which NMR data are continuously reanalyzed, reinterpreted, and integrated with orthogonal information sources.

NMR will thus evolve into a truly integrative science, bridging the gap between atomic-level observation and systems-level understanding. In chemistry, it will accelerate the discovery of new molecular architectures and reaction mechanisms. In biology, it will deepen our comprehension of molecular dynamics and disorder. In materials science, it will reveal the local structural signatures governing function and performance.

From the first detection of resonance in bulk liquids to the current frontier of in-cell and in vivo applications, NMR spectroscopy has demonstrated a remarkable capacity for renewal. Its enduring strength lies in its combination of fundamental physical rigor and methodological flexibility. As new technologies and analytical paradigms emerge, NMR adapts, incorporates, and enriches them—remaining a cornerstone of molecular science. The next generation of NMR research will likely blur traditional disciplinary boundaries, merging physical chemistry, data science, and systems biology. The ultimate goal is not simply to solve structures, but to understand how structure, dynamics, and environment give rise to function. In this sense, the evolution of NMR structural elucidation mirrors the evolution of science itself—from isolated observation toward interconnected understanding. NMR’s journey is far from over; rather, it stands poised to illuminate the molecular world with unprecedented clarity, precision, and depth.

To finish, we would like to quote Prof. James Keeler, from a 2017 interview for Magnetic Resonance in Chemistry conducted during the SMASH NMR conference, in which he was asked about the future of NMR. Although the question—and his answer—were not specifically in the context of structure elucidation, we still find the latter highly relevant and insightful [96]. “It is very hard to predict the future; you don’t see things coming. I mean, if you could see things coming you would make a lot of money. I think the thing is—and it’s always been in NMR, and I think it remains the same—it’s the technology, and the theory and the applications. They just feed on one another all the while. You get someone who wants to do something new, then some enabling technology comes along, then suddenly you can do it, and you think; if we can do that, we can do something else. That’s always been the way it is, and I think that it is always going to continue. There doesn’t seem to be any limit. It’s amazing, people have predicted the demise of NMR for years but it’s not true, it’s never happened and I don’t think it is going to happen.”

## Data Availability

The original contributions presented in this study are included in the article. Further inquiries can be directed to the corresponding author.
